# Addendum: Observation of an anti-PT-symmetric exceptional point and energy-difference conserving dynamics in electrical circuit resonators

**DOI:** 10.1038/s41467-019-10358-y

**Published:** 2019-05-30

**Authors:** Youngsun Choi, Choloong Hahn, Jae Woong Yoon, Seok Ho Song

**Affiliations:** 1 Department of Physics, Hanyang University, 222 Wangsimni-Ro, Seoul, 04763 Korea; 2 School of Electrical Engineering and Computer Science, University of Ottawa, 800 King Edward Avenue, Ottawa, ON K1N 6N5 Canada; 3 Electronics and Telecommunications Research Institute, Daejeon, 34129 Korea

**Keywords:** Microwave photonics, Optical physics, Electronic and spintronic devices

Addendum to: *Nature Communications* 10.1038/s41467-018-04690-y, published online 05 June 2018.

In this Article, we describe that a PT-symmetric Hamiltonian *H*^(PT)^ and anti-PT-symmetric Hamiltonian *H*^(APT)^ are related by a similarity transformation with a unitary operator *U* given in Eq. (7). This statement may cause confusion or incorrect arguments due to its mathematical validity limitation. Therefore, here we provide an additional explanation. This relation is strictly valid only if *H*^(PT)^ and *H*^(APT)^ are traceless and off-diagonal elements in *H*^(PT)^ are purely real. In case of non-traceless cases, one can employ an energy-shifting gauge transformation that renders *H*^(PT)^ and *H*^(APT)^ traceless, in order to make valid use of the relation. In addition, a similarity relation between traceless *H*^(PT)^ and *H*^(APT)^ can be extended for *H*^(PT)^ with complex-valued off-diagonal elements by a generalized unitary-transformation operator

$$U\prime = \frac{1}{{\sqrt 2 }}\left[ {\begin{array}{*{20}{l}} 1 \hfill & {{\mathrm{{e}}}^{i\theta }} \hfill \\ {{\mathrm{{e}}}^{ - i\theta }} \hfill & { - 1} \hfill \end{array}} \right],$$

where *θ* is phase angle of an off-diagonal element of a traceless *H*^(PT)^.

