# Supplementary material for: Observation of an anti-PT-symmetric exceptional point and energy-difference conserving dynamics in electrical circuit resonators
Source: Nat Commun. 2018 Jun 5;9:2182. doi: 10.1038/s41467-018-04690-y (PMC5988699; doi:10.1038/s41467-018-04690-y)
Supplement: Supplementary file 1 — Supplementary Information [file 41467_2018_4690_MOESM1_ESM.pdf]

Supplementary Information

Observation of an anti-*PT*-symmetric exceptional point and energy-difference conserving dynamics in electrical circuit resonators

Youngsun Choi[1,†], Choloong Hahn[2,†], Jae Woong Yoon[1,3,†,*], and Seok Ho Song[1,*]

[1]Department of Physics, Hanyang University, 222 Wangsimni-Ro, Seoul 04763, Korea.

[2]School of Electrical Engineering and Computer Science, University of Ottawa, 800 King Edward Avenue, Ottawa K1N 6N5, Ontario, Canada.

[3]Electronics and Telecommunications Research Institute, Daejeon 34129, Korea.

[*]Corresponding author: jaeong.yoon@gmail.com, shsong@hanyang.ac.kr.

Supplementary Note 1: A binary Hamiltonian model

Applying the Kirchhoff's circuit laws for the proposed model circuit in Fig. 2a in the main text, we use a simplified circuit diagram shown below. With the current-flow configuration therein, the Kirchhoff's circuit laws yield a set of equations

$$V_n = -L_n \frac{dI_{Ln}}{dt} = -\frac{1}{C_n}\int_0^t I_{Cn}(\tau)d\tau = R_n I_{Rn}\ , \tag{1}$$

$$V_1 - V_2 = R_C I_C\ , \tag{2}$$

$$I_C = I_{L1} + I_{C1} + I_{R1} = -(I_{L2} + I_{C2} + I_{R2})\ , \tag{3}$$

where n denotes a resonator index 1 or 2, I_{Ln}, I_{Cn}, and I_{Rn} are electrical current at inductor L_n, capacitor C_n, and negative-resistor unit $-R_n$, respectively. Note the sign of the electrical current takes (+) sign for the direction indicated in the simplified circuit diagram. Supplementary Equations (1) ~ (3) result in a coupled differential equations

$$\frac{d^2V_1}{dt^2} + \frac{1}{C_1}\left(\frac{1}{R_C} - \frac{1}{R_1}\right)\frac{dV_1}{dt} + \frac{1}{L_1C_1}V_1 = \frac{1}{R_CC_1}\frac{dV_2}{dt}, \tag{4}$$

$$\frac{d^2V_2}{dt^2} + \frac{1}{C_2}\left(\frac{1}{R_C} - \frac{1}{R_2}\right)\frac{dV_2}{dt} + \frac{1}{L_2C_2}V_2 = \frac{1}{R_CC_2}\frac{dV_1}{dt}. \tag{5}$$

Including the conditions $R_C = R_1 = R_2 = R$ and $C_1 = C_2 = C$ required for the APT-symmetric environmental-interaction scheme, these equations reduce to

$$\frac{d^2V_1}{dt^2} + \omega_1^2 V_1 = \alpha\frac{dV_2}{dt}, \tag{6}$$

$$\frac{d^2V_2}{dt^2} + \omega_2^2 V_2 = \alpha\frac{dV_1}{dt}\ , \tag{7}$$

where $\omega_n \equiv (L_nC)^{-1/2}$ is uncoupled-resonance angular frequency and $\alpha \equiv (RC)^{-1}$ is resistive-coupling constant. In the weak-coupling and small-detuning regimes satisfying $\alpha << \omega_n$ and $|\omega_1 - \omega_2| << 2^{-1}(\omega_1 + \omega_2)$, we further simplify Eqs. (6) and (7) with a slowly-varying complex-envelope function $v_n(t)$ such that

$$2V_n(t) = v_n(t)\exp(-i\omega_0 t) + v_n^*(t)\exp(i\omega_0 t), \tag{8}$$

where $\omega_0 \equiv 2^{-1}(\omega_1 + \omega_2)$ is average uncoupled-resonance angular frequency. Applying Supplementary Eq. (8) to Supplementary Eqs. (6) and (7), we obtain a Schrödinger-type coupled-mode equation for v_n as

$$\frac{d}{dt}\begin{bmatrix} v_1(t) \\ v_2(t) \end{bmatrix} \approx -i\frac{1}{2}\begin{bmatrix} \omega_1 - \omega_2 & i\alpha \\ i\alpha & \omega_2 - \omega_1 \end{bmatrix}\begin{bmatrix} v_1(t) \\ v_2(t) \end{bmatrix}. \tag{9}$$

Therefore, the characteristic parameters of $\mathbf{H}^{(\mathrm{APT})}$ in Eq. (2) in the main text are determined by the following relations

$$\varepsilon = 0.5(\omega_2 - \omega_1),\ \gamma = 0,\ \text{and}\ \kappa = 0.5\alpha. \tag{10}$$

The complex-envelope function $v_n(t)$ in this coupled-mode formulation allows a straightforwd interpretation of the corresponding state vector $|v\rangle = [v_1\ v_2]^{\mathrm{T}}$ as representing system's excitation amplitude and phase configurations with the inner product $\langle v|v\rangle$ being the total energy stored in the two resonators. The cycle-average total energy $\langle T_n\rangle$ for resonator n is given by a summation of the cycle-average electric energy $\langle E_n\rangle = \langle 2^{-1}C_nV_n^2\rangle$ and magnetic energy $\langle M_n\rangle = \langle 2^{-1}L_nI_{Ln}^2\rangle$, where $\langle\cdots\rangle$ indicate a time average of its argument over an oscillation cycle at ω_0. Taking into account current-flow characteristics $I_C \approx I_{Rn}$ and $I_{Cn} \approx -I_{Ln}$ in our case treating resonant behavior involving divergent impedance of the LC loop, we obtain an expression for the cycle-average total energy as

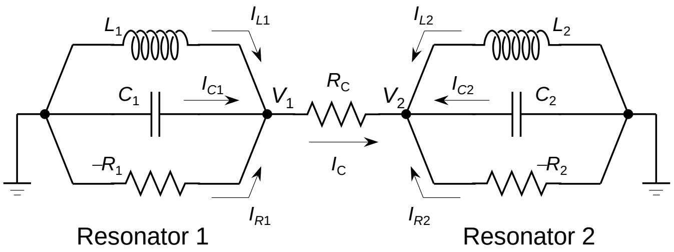

Supplementary Fig. 1 Simplified anti-parity-time-symmetric circuit diagram. Inductance, capacitance, and resistance of the circuit elements in Resonator n are denoted by L_n, C_n, and $-R_n$, respectively, with I_{Ln}, I_{Cn}, and I_{Rn} being the electric current through these elements in the same order. R_C and I_C indicate resistance of the coupling resistor and electric current through it, respectively.

$$\begin{aligned}\langle T_n\rangle &= \frac{1}{2}\langle C_nV_n^2\rangle + \frac{1}{2}\langle L_nI_{Ln}^2\rangle \\ &\approx \frac{C_n}{2}\langle V_n^2\rangle + \frac{L_nC_n^2}{2}\left\langle\left(\frac{dV_n}{dt}\right)^2\right\rangle \\ &\approx \frac{C_n}{4}|v_n|^2 + \frac{\omega_0^2}{\omega_n^2}\frac{C_n}{4}|v_n|^2 \\ &\approx \frac{C_n}{2}|v_n|^2\ .\end{aligned} \tag{11}$$

This relation provides the physical implication of the APT-symmetric conserved-quantity inner product of Eq. (6) in the main text.

Supplementary Note 2: Energy-variation rate of the APT-symmetric LRC circuit

In the APT-symmetric circuit, time derivative of the instantaneous total energy for resonator n is written by

$$\begin{aligned}\frac{dT_n}{dt} &= \frac{d}{dt}\left(\frac{1}{2}CV_n^2 + \frac{1}{2}L_nI_{Ln}^2\right) \\ &= CV_n\frac{dV_n}{dt} + L_nI_{Ln}\frac{dI_{Ln}}{dt} \\ &= V_n\left(C\frac{dV_n}{dt} - I_{Ln}\right).\end{aligned} \tag{12}$$

Using Supplementary Eqs. (1) ~ (3) in Supplementary Note 1, Supplementary Eq. (12) is further simplified as

$$\begin{aligned}\frac{dT_n}{dt} &= V_n\left(C\frac{dV_n}{dt} - I_{Ln}\right) \\ &= -V_n\left(I_{Cn} + I_{Ln}\right) \\ &= \begin{cases} -V_1(I_C - I_{R1}) & \text{for } n = 1 \\ V_2(I_{R2} + I_C) & \text{for } n = 2 \end{cases} \\ &= \begin{cases} -V_1\left(\dfrac{V_1 - V_2}{R_C} - \dfrac{V_1}{R_1}\right) & \text{for } n = 1 \\ V_2\left(\dfrac{V_2}{R_2} + \dfrac{V_1 - V_2}{R_C}\right) & \text{for } n = 2 \end{cases} \\ &= \frac{V_1V_2}{R}\quad (\because R_C = R_1 = R_2 = R).\end{aligned} \tag{13}$$

Therefore, Eq. (4) in the main text is derived for the instantaneous total energy T_n.
